# Functional Domains of ZFP809 Essential for Nuclear Localization and Gene Silencing

**DOI:** 10.1371/journal.pone.0139274

**Published:** 2015-09-29

**Authors:** Yu Ichida, Yuko Utsunomiya, Toru Yasuda, Kazuhiko Nakabayashi, Toshinori Sato, Masafumi Onodera

**Affiliations:** 1 Department of Human Genetics, National Center for Child Health and Development, 2-10-1 Okura, Setagaya, Tokyo, 157–8535, Japan; 2 Department of Maternal-Fetal Biology, National Research Institute for Child Health and Development, 2-10-1 Okura, Setagaya, Tokyo, 157–8535, Japan; 3 Department of Biosciences and Informatics, Keio University, Yokohama, Kanagawa, 223–8522, Japan; Colorado State University, UNITED STATES

## Abstract

Zinc finger protein 809 (ZFP809) is a member of the Kruppel-associated box-containing zinc finger protein (KRAB-ZFP) family, and is highly expressed in mouse immature cells. ZFP809 is known to inhibit the expression of transduced genes driven by Moloney murine leukemia virus (MoMLV)-typed retroviral vectors by binding to the primer binding site (PBS) located downstream of the MLV-long terminal repeat (LTR) of the vectors and recruiting protein complexes that introduce epigenetic silencing marks such as histone modifications and DNA methylation at the MLV-LTR. However, it remains undetermined what domains of ZFP809 among the KRAB domain at N-terminus and the seven zinc fingers are critical for gene silencing. In this study, we assessed subcellular localization, gene silencing ability, and binding ability to the PBS of a series of truncated and mutated ZFP809 proteins. We revealed the essential role of the KRAB A box for all functions assessed, together with the accessory roles of a subset of zinc fingers. Our data also suggest that interaction between KAP1 and the KRAB A box of ZFP809 is critical in KAP1-dependent control of gene silencing for ZFP809 targets.

## Introduction

Kruppel-associated box-containing zinc finger proteins (KRAB-ZFPs) comprises the largest single family of transcription factors in mammals and are found only in tetrapod vertebrates [1,2]. Zinc finger motifs within KRAB-ZFPs bind to specific DNA sequences and regulate the transcription of target genes, functioning together with KRAB-associated protein 1 (KAP1) [3–6]. Although previous studies have reported the involvement of KRAB-ZFPs in various cellular processes such as metabolism, immunity, apoptosis, cancer, development and differentiation [1,4,7–10], cellular functions and subcellular localization of many KRAB-ZFPs remain unknown. A recent study has shown that ZNF268, a member of human KRAB-ZFPs, is localized in the nucleus and not nucleoli through cooperative functions of zinc finger motifs and the KRAB domain [11].

In 2009, Wolf and Goff revealed that zinc finger protein 809 (ZFP809) has a central role in the transcriptional suppression of Moloney murine leukemia virus (MoMLV) [12]. Furthermore, ZFP809 has also been shown to be required to initiate silencing of endogenous retroviruses (ERVs) during mouse embryonic development [13].

ZFP809 contains the KRAB domain at the N-terminus and seven zinc fingers at the C-terminus. ZFP809 is highly expressed in immature cells such as embryonic stem cells [12] and may block the horizontal transmission of retrovirus from immature cells to mature cells. ZFP809 binds to the primer binding site for proline tRNA (PBS-Pro sequence) located downstream of the 5' long terminal repeat (LTR), and the KRAB domain within ZFP809 that interacts with KAP1, which further recruits the ERG-associated protein with SET domain (ESET, a H3K9 methyltransferase), heterochromatin protein 1 (HP1) and the NuRD histone deacetylase complex. The resultant protein complex represses retroviral gene expression by inducing epigenetic modifications for an inactive chromatin state [14,15]. Thus, ZFP809 inhibits the expression of transduced genes driven by lentiviral or retroviral vectors containing the MLV PBS-Pro sequence.

Although ZFP809 is one of the most well-characterized KRAB-ZFPs, it remains unclear which of the seven zinc fingers of ZFP809 are responsible for binding to the PBS and the ability to silence MLV-LTR-driven gene expression. Furthermore, while ZFP809 is considered to be localized in the nucleus because it represses transgene expression driven by MLV-LTR [12] and silences ERVs [13], the subcellular localization of ZFP809 has not been directly assessed in the previous studies [12, 13]. Therefore, in this study, we addressed these issues by constructing vectors to exogenously express ten types of truncated/mutated ZFP809 proteins in cultured cells, and investigated their sub-nuclear localization, gene silencing efficiency and binding ability to the PBS.

## Materials and Methods

### Cell culture

The 293FT cell line was purchased from Life technologies and was cultured in Dulbecco’s Modified Eagle’s Medium (DMEM) supplemented with 10% fetal calf serum (FCS, Thermo scientific), 2 mM L-glutamine, 0.1 mM sodium pyruvate, 100 U/mL penicillin G sodium, and 100 μg/mL streptomycin sulfate, at 37°C in 5% CO_2_. 293gpg cells were maintained in DMEM (Sigma-Aldrich), 10% heat-inactivated FBS (Sigma-Aldrich) supplemented with 0.3 mg/mL G418 (Sigma-Aldrich), 2 μg/mL puromycin (Sigma-Aldrich), and 1 μg/mL tetracycline (Sigma-Aldrich), at 37°C in 10% CO_2_.

### Vector construction

The sequences of oligo DNAs used for vector construction (PCR primers, mutagenic primers, and DNA oligos for shRNA vector preparation) are shown in S1 Table. The coding sequence of *Zfp809* was amplified by reverse-transcription PCR using total RNA extracted from F9 cells. The PCR fragments were sub-cloned into pCR2.1 TA cloning vector (The Original TA cloning Kit, Life Technologies). The resultant plasmid DNA was digested with *NotI* and *EcoRV*, and the fragments were inserted into *NotI*/*EcoRV*-digested p3×FLAG-CMV-10 vector (Sigma-Aldrich). The resultant plasmid DNA was referred to as pCMV/flag-Zfp809.

Nucleotide substitutions were introduced in pCMV/flag-Zfp809 using the PrimeSTAR Mutagenesis Basal Kit (TAKARA BIO) with the mutagenic primers (S1 Table) so that amino acid substitutions of E13A, E14A and W15A were introduced in the KRAB domain. The resultant vector was referred to as pCMV/flag-mtKRAB. cDNAs for truncated forms of ZFP809 were amplified using pCMV/flag-Zfp809 as template DNA with primer sets listed in S1 Table. The resultant PCR fragments were digested with *NotI* and *BamHI*, and inserted into *NotI*/*BamHI*-digested p3×FLAG-CMV-10 vector. These plasmid vectors, taken together with pCMV/flag-mtKRAB, were referred to as pCMV/flag-X (X denotes any of: intact ZFP809, ΔSD, ΔZF, ZF1, ZF1-2, ZF1-3, ZF1-4, ZF1-5, ZF1-6, ΔKRAB_A, or mtKRAB).

To prepare lentiviral vectors, pLVSIN/IRES/ZsGreen vector (TAKARA BIO) was used as a backbone vector. A *MluI* site was introduced in the vector using the PrimeSTAR Mutagenesis Basal Kit using mutagenic primers listed in S1 Table. *Mlu*I fragments containing mCherry cDNA were inserted into the *Mlu*I site. The resultant vector was referred to as pLVSIN/IRES/mCherry vector. cDNAs encoding intact, truncated and mutated ZFP809 were amplified using one of the pCMV/flag-Zfp809, pCMV/flag-ΔKRAB or pCMV/flag-mtKRAB as a template DNA with primers listed in S1 Table, digested with *EcoRI* and *BamHI*, and inserted into *EcoRI*/*BamHI*-digested pLVSIN/IRES/mCherry vector. The resultant lentiviral vectors were referred to as pLVSIN/CMV/flag-X/IRES/mCherry (X denotes any of: intact ZFP809, ΔSD, ΔZF, ZF1, ZF1-2, ZF1-3, ZF1-4, ZF1-5, ZF1-6, ΔKRAB_A, or mtKRAB). To generate lentiviruses, each of the pLVSIN/CMV/flag-X/IRES/mCherry vectors was co-transfected with packaging vectors (ViraPower packaging Mix, Life Technologies) into 293FT cells using Lipofectamine LTX (Life Technologies). Culture supernatants were harvested on day 1, 2 and 3 and transduced to the target cells with 4 μg/mL of polybrene by spinoculation of 1,000 *g* for 1 hour at 32°C [16].

To prepare retroviral vectors, *Nco*I and *Cla*I fragments containing EGFP cDNA were cloned into the retroviral vector GCsapMLV carrying the MLV-derived PBS [17]. The retroviral vectors were converted to the corresponding retroviruses packaged in VSV-G envelop by transfecting the vectors into 293GPG cells. The resultant retroviruses were then transduced to the target cells with 4 μg/mL of polybrene by spinoculation of 1,000 *g* for 1 hour at 32°C [16].

To prepare shRNA vectors for KAP1 and negative control (NC), *Nco*I and *Cla*I fragments containing EGFP cDNA were cloned into *BamH*I/*Kpn*I-digested pLKO.1 vector (Thermo Scientific Open Biosystems Expression Arrest). An *AgeI* site was added to the pLKO.1 vector containing EGFP cDNA using the PrimeSTAR Mutagenesis Basal Kit with the mutagenic primers listed in S1 Table. DNA oligos corresponding to shRNA for negative control (NC) and KAP1, designed using the siDirect website (http://sidirect2.rnai.jp/), were inserted into the *Age*I/*EcoR*I-digested pLKO.1 vector. The sequences of DNA oligos are listed in S1 Table.

For luciferase reporter assay, pGL4.50 vector (Promega) was inserted with the MLV- or dl587rev-derived PBS at downstream of the CMV promoter, and referred to as pCMV_MLV/luc or pCMV_dl587/luc, respectively.

### Immunostaining

Each of the pLVSIN/CMV/flag-X/IRES/mCherry vectors was transduced with 293FT cells. The transduced cells were cultured on the chamber slide (Thermo Scientific Nunc Lab-Tek II CC2), fixed with 4% paraformaldehyde (WAKO) for 30 min at 4°C, washed with buffer containing phosphate buffered saline (PBS) and 0.05% (v/v) Triton X-100 three times, and permeabilized with buffer containing PBS and 0.5% (v/v) Triton X-100 for 15 min at room temperature. Subsequently, the slides were blocked with buffer containing PBS, 0.05% (v/v) Triton X-100 and 10% Block Ace (DS PHARAMA BIOMEDICAL) for 60 min at room temperature and incubated with the anti-FLAG monoclonal antibody (F1804, Sigma-Aldrich), anti-KAP1 polyclonal antibody (ab10484, Abcam) or anti-Fibrillarin polyclonal antibody (#2639, Cell Signaling) as a primary antibody in buffer containing PBS, 0.05% (v/v) Triton X-100 and 2% Block Ace for overnight. Next, the slides were washed with buffer containing PBS and 0.05% (v/v) Triton X-100 three times and incubated with Cy5-donkey anti-rabbit IgG or Cy3-donkey anti-mouse IgG as a secondary antibody (Jackson ImmunoResearch) for 60 min at room temperature. The slides were washed with buffer containing PBS and 0.05% (v/v) Triton X-100 three times and stained with DAPI in the presence of VECTASHIELD Mounting Medium (Vector Laboratories). Fluorescent images were captured suing an IX81 microscope (Olympus) with the FV1000 configuration. The scale bars included in the images represent 10 μm. Three independent experiments were performed to assess the subcellular localization of truncated and mutated ZFP809 proteins.

### Immunoprecipitation and Western Blotting

Cell lysates were extracted from 293FT cells transduced with one of the pLVSIN/CMV/flag-X/IRES/mCherry vectors using the extraction buffer (5 × IP buffer (Life technologies), 20 mM NaCl, 2 mM MgCl2, 1 mM DTT). The anti-KAP1 polyclonal (ab10484, Abcam) or the anti-FLAG monoclonal antibody (F1804, Sigma-Aldrich) was conjugated with Dynabeads protein G (Life Technologies) according to manufacturer’s instructions, and was used for immunoprecipitation The cell lysates and each of the antibodies were incubated for 15 min at room temperature. The immunoprecipitated samples were electrophoresed on 4–20% Mini-PROTEAN TGX Precast Gels (BIORAD) and transferred to a polyvinylidene fluoride (PVDF) membrane using the Trans-Blot Turbo Mini PVDF Transfer Packs (BIORAD). The PVDF membrane was blocked with Blocking One (NACALAI TESQUE) for 30 min at room temperature. Subsequently the PVDF membrane was incubated overnight at 4°C with the anti-DDDDK-tag mAb-HRP-DirecT (MBL), the anti-KAP1 monoclonal (ab22553, Abcam) or polyclonal (ab10484, Abcam) antibodies (1:1000 dilution in Tris-buffered saline-Tween (TBST) solution (containing 1x TBS and 0.1% Tween 20)/Blocking One (1:20)). The anti-DDDDK tag antibody is equivalent to FLAG antibodies from Sigma-Aldrich. The membrane was washed in TBST for 5 min and then incubated with the horseradish peroxidase conjugated goat anti-mouse or rabbit IgG secondary antibody (GE Healthcare Life Sciences, 1:5000 dilution in TBST/Bloking One) for 2 h at room temperature. After a final wash with TBST for 5 min, signals were detected using the ECL system (GE Healthcare Life Sciences).

### Luciferase reporter assay

293FT cells were seeded at 25,000 cells per well in a 96-well plate and were transfected using Lipofectamine LTX with 10 ng of pCMV/MLV/Luc or pCMV/dl587/Luc, 10 ng of a TK promoter-driven Renilla luciferase control plasmid vector (pGL4.74, Promega), and 45 ng of one of the pCMV/flag-X vectors. Luciferase activity was assayed at 24 h after transfection using dual-luciferase reporter assay kit (Promega). The ratio of firefly luciferase activity to renilla luciferase activity was calculated and presented as relative response ratio (RRR). Statistical analyses were performed with Bonferroni correction.

### Flow cytometry analysis

Cells were washed, resuspended with PBS containing 2% FCS and analyzed or sorted based on EGFP or mCherry expression using FACSAria™ III (BD Biosciences).

### Nuclear extracts and Electrophoretic Mobility Shift Assay (EMSA)

Nuclear extracts were obtained from 293FT cells transduced with the pLVSIN/CMV/flag-X/IRES/mCherry vectors using Nuclear/Cytosol Fractionation Kit (BioVision). The sense sequence of the oligonucleotide probe containing the MLV-PBS sequence (undermined) is 5′- TTTGGGGGCTCGTCCGGGATTT -3’.

Double-stranded probes were prepared by annealing the sense and the antisense single-stranded oligonucleotides, end-labeled with [γ-32P]dATP (PerkinElmer) using T4 polynucleotide kinase (New England Biolabs), and purified with MicroSpin G-25 Columns (GE Healthcare). Ten microliters of nuclear extracts were mixed with 20 μL binding buffer (100 mM HEPES pH7.9, 250 mM KCL, 5 mM EDTA, 5 mM dithiothreitol, 15 mM MgCl2, 5% glycerol) containing 3 μL FCS, 2 μL poly (dI/dC) (Sigma-Aldrich) and incubated for 20 min at room temperature. One microliter of the purified probes was added in the reaction mix and incubated for 20 min at room temperature. The anti-FLAG monoclonal antibody (F1804, Sigma-Aldrich) or the anti-KAP1 monoclonal antibody (ab22553, Abcam) was added for super-shift. Excessive cold probes were added as competitor. The samples were electrophoresed on 5% native acrylamide gels, dried, and visualized with a BAS phosphor screen.

### Establishment of KAP1-knockdown cells

293FT cells were transduced with lentivirus generated from shRNA vectors for KAP1 or negative control (NC), and sorted based on EGFP expression using the FACSAria™ III. The knockdown efficiency of KAP1 in the sorted cells was confirmed by Western blot analysis using the anti-KAP1 monoclonal antibody (ab22553, Abcam) and the Anti-β-Actin pAb-HRP-DirecT (MBL). The sorted 293FT cells expressing shRNA for KAP1 were transduced with the pLVSIN/CMV/*flag-*Zfp809/IRES/mCherry vector, or pLVSIN/CMV/flag-KRAB_A/IRES/mCherry vector, and sorted based on EGFP and mCherry expression using the FACSAria™ III. The sorted cells were subjected to the EMSA experiment.

## Results and Discussion

### Truncated and mutated forms of ZFP809 proteins expressed from a lentiviral vector

**Fig 1:**
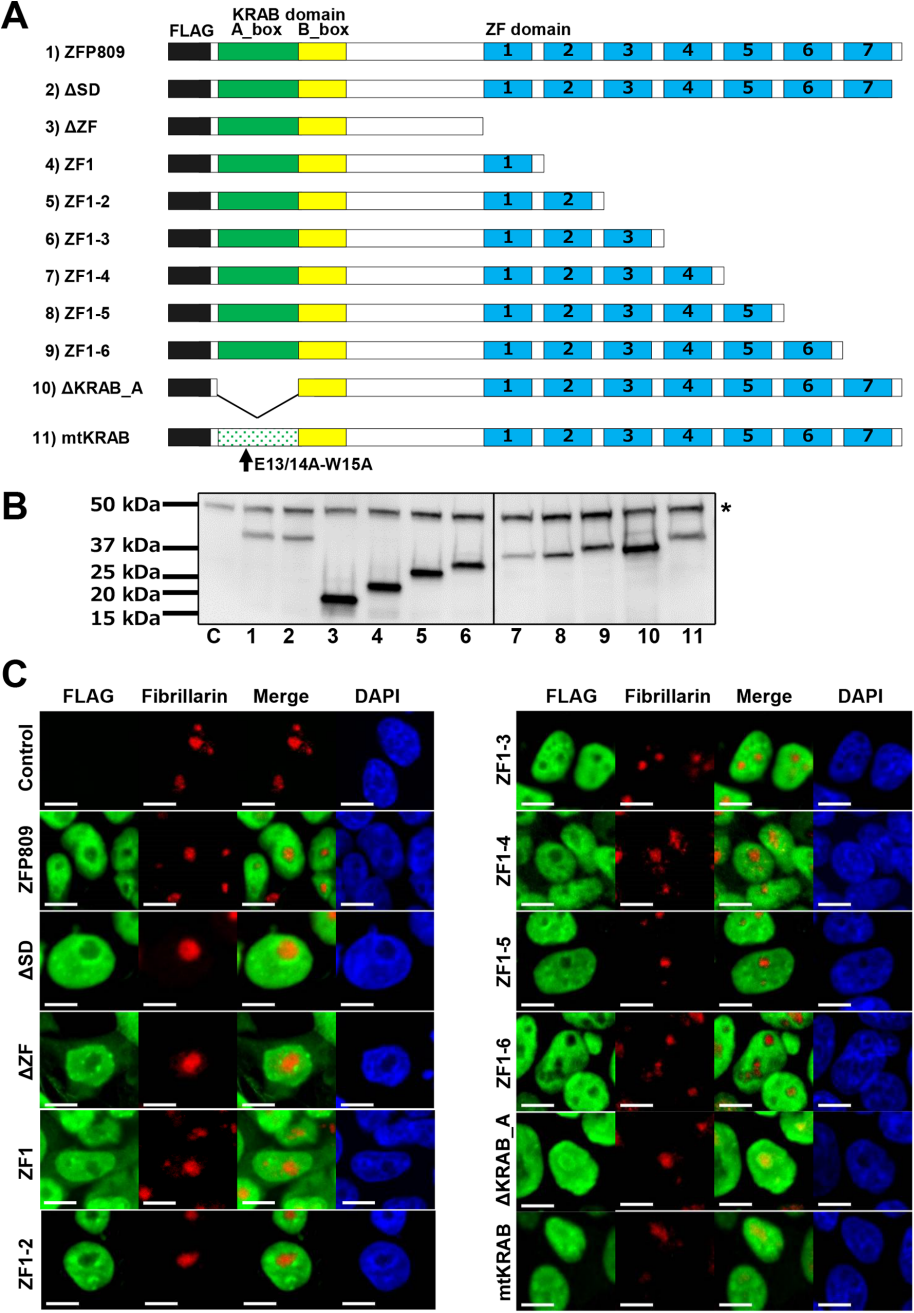
Domain structures and subcellular localization of intact, truncated, and mutated ZFP809 proteins. (A) Schematic representation of the domain structures of the intact ZFP809 protein (1), a series of truncated proteins (2–10), and a mutated ZFP809 protein with three amino acid substitutions within the KRAB domain (11), assessed for their functionalities in this study. ZF and SD denote zinc finger and spacer domain, respectively. All constructs were attached with the FLAG tag at the N-terminus and cloned into the pLVSIN/IRES/mCherry vector (S1A Fig), and referred to as pLVSIN/CMV/flag-X/ IRES/mCherry vectors (X denotes any of: intact ZFP809, ΔSD, ΔZF, ZF1, ZF1-2, ZF1-3, ZF1-4, ZF1-5, ZF1-6, ΔKRAB_A, or mtKRAB). ΔSD lacks a spacer domain of six amino acids at the C-terminus of ZFP809. (B) Confirmation of the size of the proteins expressed from the eleven vector constructs shown in Fig 1A. 293FT cells transduced with one of the eleven vector constructs were sorted based on mCherry expression. The sorted cells were subjected to Western blot analysis using anti-FLAG antibody (lane C, empty vector (pLVSIN/CMV/flag/IRES/mCherry); lanes 1 to 11, eleven vector constructs). The asterisk indicates non-specific bands. (C) Sub-nuclear localization of intact and truncated/mutated ZFP809 proteins exogenously expressed from lentiviral vectors in 293FT cells detected by immunostaining using the anit-FLAG antibody followed by confocal microscopic analysis. 293FT cells transduced with the pLVSIN/CMV/flag/IRES/mCherry vector were used as the “Control”. Anti-fibrillarin antibody was also used as a nucleolus marker. Two single-stained, merged, and DAPI-stained images are shown.

ZFP809 contains two major domains, one KRAB domain and a domain of seven zinc fingers. As a tool to identify functionally important sub-domains in ZFP809, we generated vector constructs for intact ZFP809 proteins, together with nine types of truncated forms and a mutated form using the lentiviral vector pLVSIN/CMV/IRES/mCherry (S1A Fig). All forms of proteins are tagged with FLAG at the N-terminus. Proteins are forcedly expressed under human cytomegalovirus (CMV) immediate early enhancer-promoter control in mammalian cells. A fluorescent protein, mCherry, is also expressed bicistronically under the control of the CMV promoter. The sub-domain structures of the nine types of truncated forms (referred to as ΔSD, ΔZF, ZF1, ZF1-2, ZF1-3, ZF1-4, ZF1-5, ZF1-6, and ΔKRAB_A) are depicted in Fig 1A. The mutated form of ZFP809 protein, referred to as mtKRAB, has amino acid substitutions at the 13^th^ to 15^th^ amino acid residues of the KRAB domain (E13/14A-W15A corresponding to E16/17A-W18A of ZNF268) (Fig 1A and S2 Fig). These amino acid substitutions have been shown to abolish the interaction between KRAB-ZFPs and KAP1 [18]. We confirmed protein expression from the 11 lentiviral vector constructs for ZFP809 proteins shown in Fig 1A by transducing 293FT cells with each of the vectors, followed by sorting mCherry expressing cells and Western blot analysis (Fig 1B). We used these vector constructs to assess the functionality of truncated/mutated forms of ZFP809 proteins in terms of nuclear localization, gene silencing efficiency, and binding ability to PBS.

### The KRAB domain and zinc fingers cooperatively control the nuclear localization pattern of ZFP809

A previous study has reported that both of the KRAB domain and zinc fingers are necessary for the nuclear localization of KRAB-ZFPs [11]. To determine sub-domains within ZFP809 responsible for its nuclear localization, we subjected 293FT cells expressing the intact or one of the truncated/mutated ZFP809 proteins to immunostaining using anti-FLAG and anti-fibrillarin antibodies, of which the latter is used to stain nucleoli. The intact ZFP809 protein was localized in the nucleus excluding the nucleoli (Fig 1C and S3A Fig), consistent with a previous study showing that no KRAB-ZFPs were detected in purified nucleoli [19]. The truncated forms, ΔSD, ZF1-2, ZF1-3, ZF1-4, ZF1-5, and ZF1-6 behaved in the same way as the intact ZFP809. Among the truncated forms of ZFP809, ΔZF and ZF1 were mainly localized in the nucleus excluding the nucleoli, but were also localized in the cytoplasm (Fig 1C and S3A Fig). ΔKRAB_A and mtKRAB were localized in the nucleus in all cells examined. However, the localization of these proteins differed from that of intact ZFP809 and the other truncated versions in that ΔKRAB_A and mtKRAB were localized in the nucleus including nucleoli in the majority (approximately 80% and 70%, respectively) of the cells examined (Fig 1C and S3A Fig). The localization of ΔKRAB_A and mtKRAB in nucleoli was suggested by the overlap in the staining by the nucleolus maker and the anti-FLAG antibody. The alteration in the nucleolar localization pattern of ZFP809 upon the truncation/mutation of its KRAB A box observed in this study is consistent with the previous report for KRAB-ZFPs other than ZFP809 [11]. These results suggest that the KRAB A box domain and the first and second zinc fingers are required for the proper nuclear localization of ZFP809.

### The KRAB domain contributes to the nuclear localization pattern of ZFP809 through its interaction with KAP1

**Fig 2:**
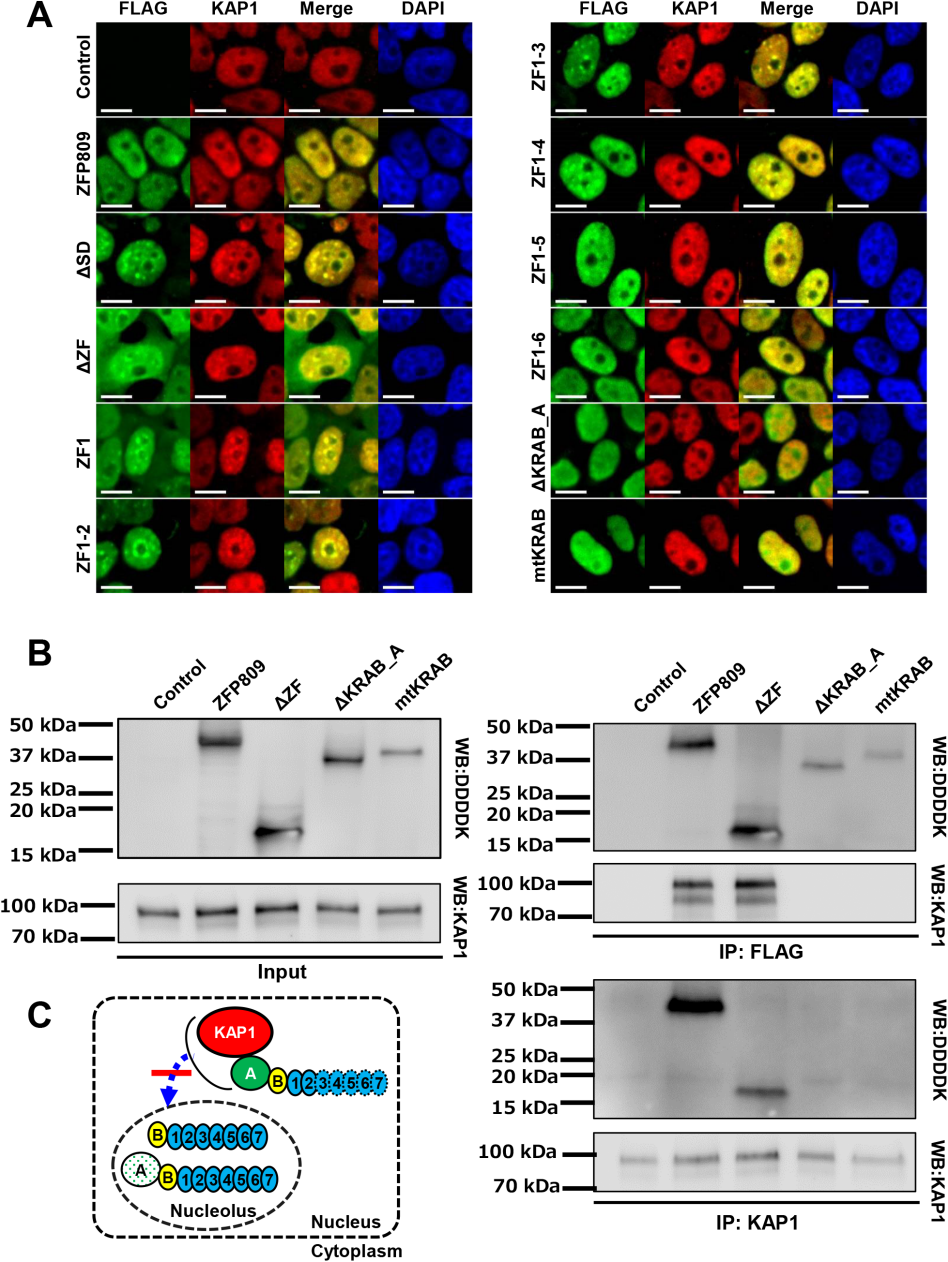
Co-localization and interaction of intact, truncated, and mutated ZFP809 proteins with KAP1. (A) 293FT cells transduced with pLVSINCMV/flag-X/IRES/mCherry vectors were immunostained with anti-FLAG antibody and anti-KAP1 antibody followed by confocal microscopic analysis. X denotes either of ZFP809, ΔZF, ZF1, ZF1-2, ZF1-3, ZF1-4, ZF1-5, ZF1-6, ΔSD, ΔKRAB_A, or mtKRAB. 293FT cells with lentiviral transduction of the pLVSIN/CMV/flag/IRES/mCherry vector were used as the “Control”. Two single-stained, merged, and DAPI-stained images are shown. (B) Immunoprecipitation (IP) followed by Western blot analysis to assess the interaction of intact, truncated and mutated ZFP809 proteins with KAP1. Protein extracts from 293FT cells that were transduced with the lentiviral vectors corresponding to ZFP809, ΔZF, ΔKRAB_A, and mtKRAB (Fig 1A). The antibody used for IP and Western blotting is shown on the left and right sides of the band images, respectively. Anti-DDDDK tag antibody is equivalent to FLAG antibodies from Sigma. (C) A schematic model of the role of KAP1 in preventing the entry of ZFP809 into nucleoli. Our results shown in Fig 2A and B suggest that interaction of KAP1 with ZFP809 through its KRAB A box domain is necessary for the proper nuclear distribution of ZFP809 being excluded from nucleoli.

Previous studies have demonstrated that the KRAB domain within KRAB-ZFPs interacts with KAP1 and reinforces nuclear localization, and that the amino acid substitutions (E16/17A-W18A of ZFP268) in the KRAB domain nullify its interaction with KAP1 [11,18]. We assessed whether each of truncated and mutated forms of ZFP809 (Fig 1A) co-localizes with KAP1 by immunostaining of 293FT cells expressing the intact ZFP809 or one of the truncated/mutated ZFP809 proteins. Irrespective of the type of ZFP809 proteins co-expressed exogenously in 293FT cells, KAP1 was found to be localized only in the nucleus excluding nucleoli (Fig 2A and S3B Fig). Consistent with their proper nuclear localization pattern (being excluded from nucleoli) shown in Fig 1C, the intact ZFP809 protein as well as the truncated forms, ΔSD, ZF1-2, ZF1-3, ZF1-4, ZF1-5, and ZF1-6 showed the same nuclear localization pattern as KAP1 in all cells assessed (Fig 2A and S3B Fig). Although ΔZF and ZF1 were localized in both the cytoplasm and the nucleus, these truncated forms also showed the same nuclear localization pattern as KAP1 (Fig 2A and S3B Fig). However, ΔKRAB_A and mtKRAB showed the same nuclear localization pattern as KAP1 only in approximately 30% and 40% respectively of the cells assessed (Fig 2A and S3B Fig).

Consistently, an interaction with KAP1 was detected for the intact ZFP809 and ΔZF but not for ΔKRAB_A and mtKRAB in 293FT cells in immunoprecipitation analysis followed by Western blot (Fig 2B). These results demonstrate that the interaction with KAP1 through the KRAB domain is essential for the proper nuclear distribution of ZFP809, specifically exclusion from nucleoli (Fig 2C).

### The KRAB domain and five zinc fingers of ZFP809 are required for the PBS-dependent gene silencing of MLV-LTR driven transgene expression

**Fig 3:**
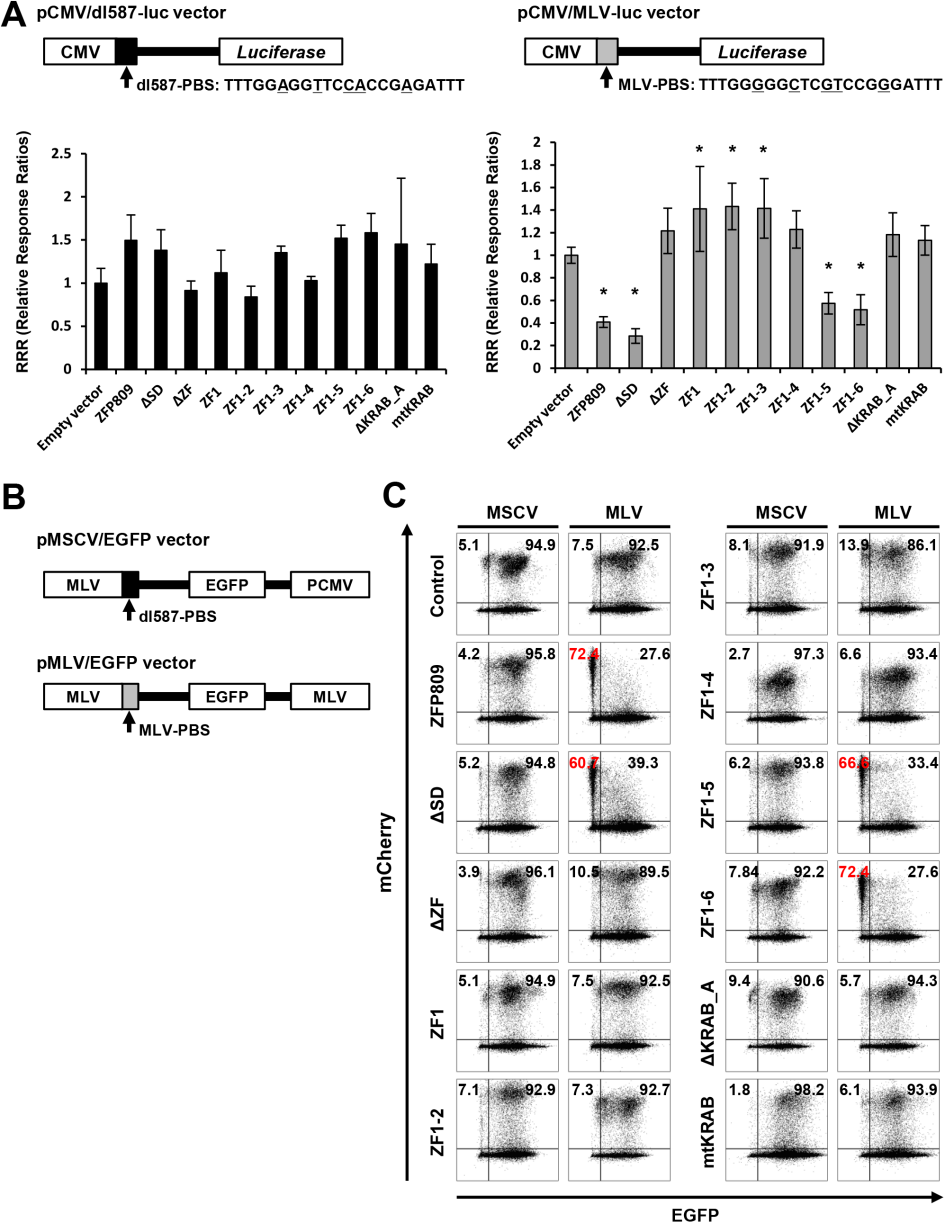
Identification of ZFP809 sub-domains required for the PBS-dependent gene silencing of MLV-LTR driven transgene expression. (A) The structures of luciferase reporter vectors used are shown. Black and grey boxes at downstream of the CMV promoter represent the MLV- and dl587rev-derived PBS, respectively. The five nucleotide positions whose base sequence is different between two PBSs are underlined. 293FT cells were co-transfected with one of the reporter vectors and one of the pCMV/flag-X vectors (X denotes either of ZFP809, ΔSD, ΔZF, ZF1, ZF1-2, ZF1-3, ZF1-4, ZF1-5, ZF1-6, ΔKRAB_A, or mtKRAB) (S1B Fig). RRR (relative response ratio) represents the value of the firefly luciferase activity relative to that of the *Renilla* luciferase activity (control) at 24 hours after transfection. Error bars show the standard deviation obtained from four independent experiments. Asterisks indicate statistical significance (*P* <0.05) with *Bonferroni* correction. (B) The structures of the retroviral GFP reporter vectors. When the RNA genome derived from retroviral vectors is reverse transcribed into an RNA-DNA duplex and then into double-stranded DNA, a part of 5' LTR is recombined to a part of 3' LTR. (C) 293FT cells transduced with one of the retroviral vectors (MLV/EGFP or MSCV/EGFP) were sorted based on EGFP expression, and transduced with one of the pLVSIN/CMV/flag-X/IRES/mCherry vectors or the control vector (pLVSIN/CMV/flag/IRES/mCherry). Then, the expression levels of EGFP and mCherry were analyzed at day 4 (S4 Fig) and day 15 (Fig 3C) after transduction.

To determine critical sub-domains of ZFP809 for silencing of a transgene driven by MLV, we subjected the series of truncated and mutated ZFP809 proteins (Fig 1A) to two types of assays to examine their silencing activity. A minimal MLV PBS sequence, GGGGGCTCGTCCGGGA (16 bp), flanked by TTT at both ends, was used in these assays. First, we carried out reporter assays using the firefly luciferase (Luc) reporters driven by the cytomegalovirus (CMV) promoter containing the MLV- or dl587rev-derived PBS immediately downstream of the CMV promoter, referred to as pCMV/MLV/Luc or pCMV/dl587/Luc, respectively. The sequence of dl587rev-derived PBS is different at five base pair positions from that of the MLV-PBS and lacks a consensus binding site for ZFP809 [20]. When 293FT cells were transfected with CMV/dl587/Luc and one of the vectors expressing truncated and mutated ZFP809 proteins (S1B Fig), the extent of the Luc activity was comparable to that of the Luc activity in 293FT cells transfected with pCMV/dl587/Luc and the vector expressing the intact ZFP809 (Fig 3A, left), indicating that none of the ZFP809 proteins tested suppressed the Luc activity. On the other hand, when 293FT cells were transfected with pCMV/MLV/Luc and one of the vectors expressing truncated and mutated ZFP809 proteins, only ΔSD, ZF1-5, and ZF1-6 showed Luc activity levels comparable to that of cells transfected with pCMV/MLV/Luc and the vector expressing the intact ZFP809 (Fig 3A, right). The relative responsive ratios (RRRs) of 293FT cells transfected with the other truncated forms and the mutated form of ZFP809 were three times higher than the RRR of cells transfected with vector expressing the intact ZFP809. These results suggest that ΔSD, ZF1-5, and ZF1-6 retain their gene silencing ability at a level similar to that of the intact ZFP809.

Furthermore, as a second type of assay, we monitored the gene silencing effect of the series of truncated/mutated ZFP809 proteins on sustained transgene expression. We transduced 293FT cells with either MLV- and MSCV-typed vectors expressing EGFP (referred to as pMLV/EGFP and pMSCV/EGFP, respectively) (Fig 3B), and sorted EGFP-expressing cells using FACS Aria. We further transduced the EGFP-expressing cells with each of the series of the pLVSIN/CMV/flag-X/IRES/mCherry vectors and flow cytometrically analyzed the fluorescent intensities of EGFP and mCherry at day 4 and 15 after transduction (Fig 3C and S4 Fig). While EGFP expression levels from the MSCV/EGFP vector were unaltered between the absence and presence of ZFP809 proteins (the first and third lines in Fig 3C), those from the MLV/EGFP vector were declined only in the presence of the intact ZFP809, ΔSD, ZF1-5, or ZF1-6 (the second and forth lines in Fig 3C). Therefore, our data from the two types of assays assessing the gene silencing ability of ZFP809 consistently indicate that the KRAB domain and the first to fifth zinc fingers are required to suppress transgene expression from the MLV-LTR.

### The first to fifth zinc fingers are required for the binding of ZFP809 to MLV-derived PBS

**Fig 4:**
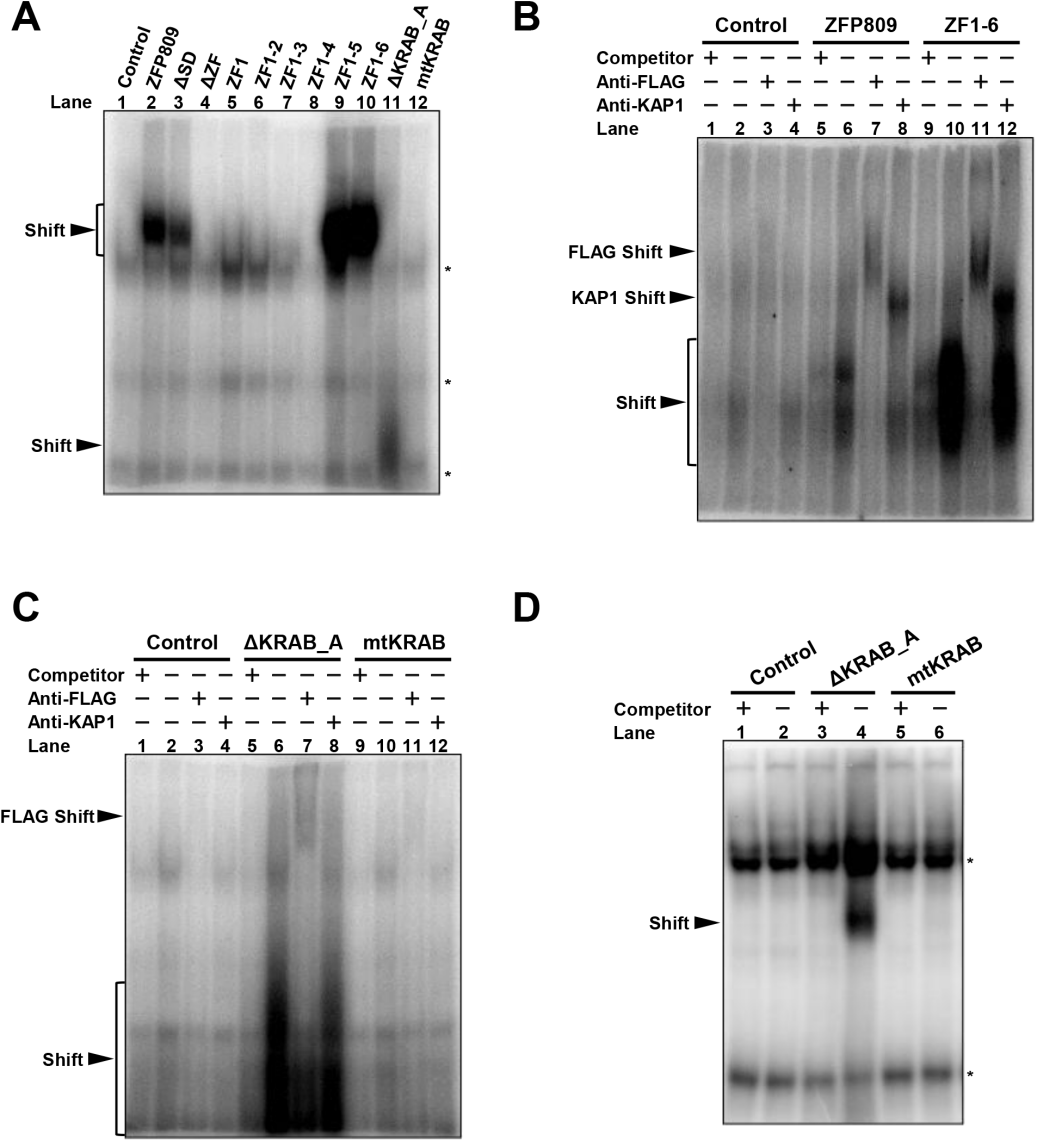
Assessment of truncated and mutated ZFP809 proteins for their binding ability to MLV-PBS. (A) EMSA of the MLV-PBS sequence with the series of intact/truncated/mutated ZFP809 proteins (8% gel). Asterisks indicate non-specific bands. (B) EMSA of the MLV-PBS sequence with the intact ZFP809 and ZF1-6 proteins in the presence of cold competitor DNA, anti-FLAG antibody, or anti-KAP1 antibody (8% gel). (C) and (D) EMSA of the MLV-PBS sequence with the ΔKRAB_A and mtKRAB proteins in the presence of cold competitor DNA, anti-FLAG antibody, or anti-KAP1 antibody electrophoresed on 8% gel (C) and 10% gel (D). Asterisks indicate non-specific bands. In “Control” lanes in A-D, the probe DNA was mixed with nuclear extracts from 293FT cells with lentiviral transduction of pLVSIN/CMV/flag/IRES/mCherry, and electrophoresed.

Since gene silencing by ZFP809 is exerted in a manner dependent of the PBS sequence [12], we next assessed whether the truncated/mutated ZFP809 proteins directly bind to the MLV-derived PBS by an electrophoretic mobility shift assay (EMSA). Nuclear extracts from 293FT cells transduced with one of the lentiviral vector constructs for the intact/truncated/mutated ZFP809 proteins (Fig 1A) were mixed with a 22-bp ^32^P-labeled probe corresponding to the MLV-PBS sequence, and then electrophoresed in 8% polyacrylamide gels. Mobility shift of the probe DNA, indicative of protein interaction with the probe, was observed for ZFP809, ΔSD, ZF1-5, and ZF1-6 (Fig 4A, lanes 2, 3, 9, and 10). Interestingly, ΔKRAB_A (lane 11), which did not silence the MLV-LTR driven transgene, showed a mobility shift at a lower position compared with ZFP809, ΔSD, ZF1-5, and ZF1-6. The difference in the extent of mobility shift is likely to be dependent on the absence or the presence of KAP1 interaction with the KRAB_A domain. The mobility shifts detected using ZFP809 and ZF1-6 (Fig 4B, lanes 6 and 10) were diminished in the presence of excess cold probe DNA as a competitor (lanes 5 and 9), and super-shifted by the addition of anti-FLAG (lanes 7 and 11) or anti-KAP1 antibody (lanes 8 and 12). As shown in Fig 4C, the bands detected for ΔKRAB_A (lane 6) were diminished by the presence of excessive cold probes (lane 5), and were partially super-shifted in the presence of anti-FLAG antibody (lane 7) but not in the presence of anti-KAP1 antibody (lane 8). These results suggest that ΔKRAB_A interacts with MLV-PBS. In contrast, no band was detected for mtKRAB (lane 10). The binding of ΔKRAB_A to MLV-PBS was also confirmed by the EMSA analysis using a 10% gel (Fig 4D). A shifted band was detected for ΔKRAB_A but not for mtKRAB.

These results suggest that ZFP809 interacts with MLV-PBS through the first to the fifth zinc fingers, and when the KRAB_A domain is unbound to KAP1, it prevents the zinc finger domain from binding to MLV-PBS (.

### Binding ability of ZFP809 to MLV-PBS is dependent of its interaction with KAP1

**Fig 5:**
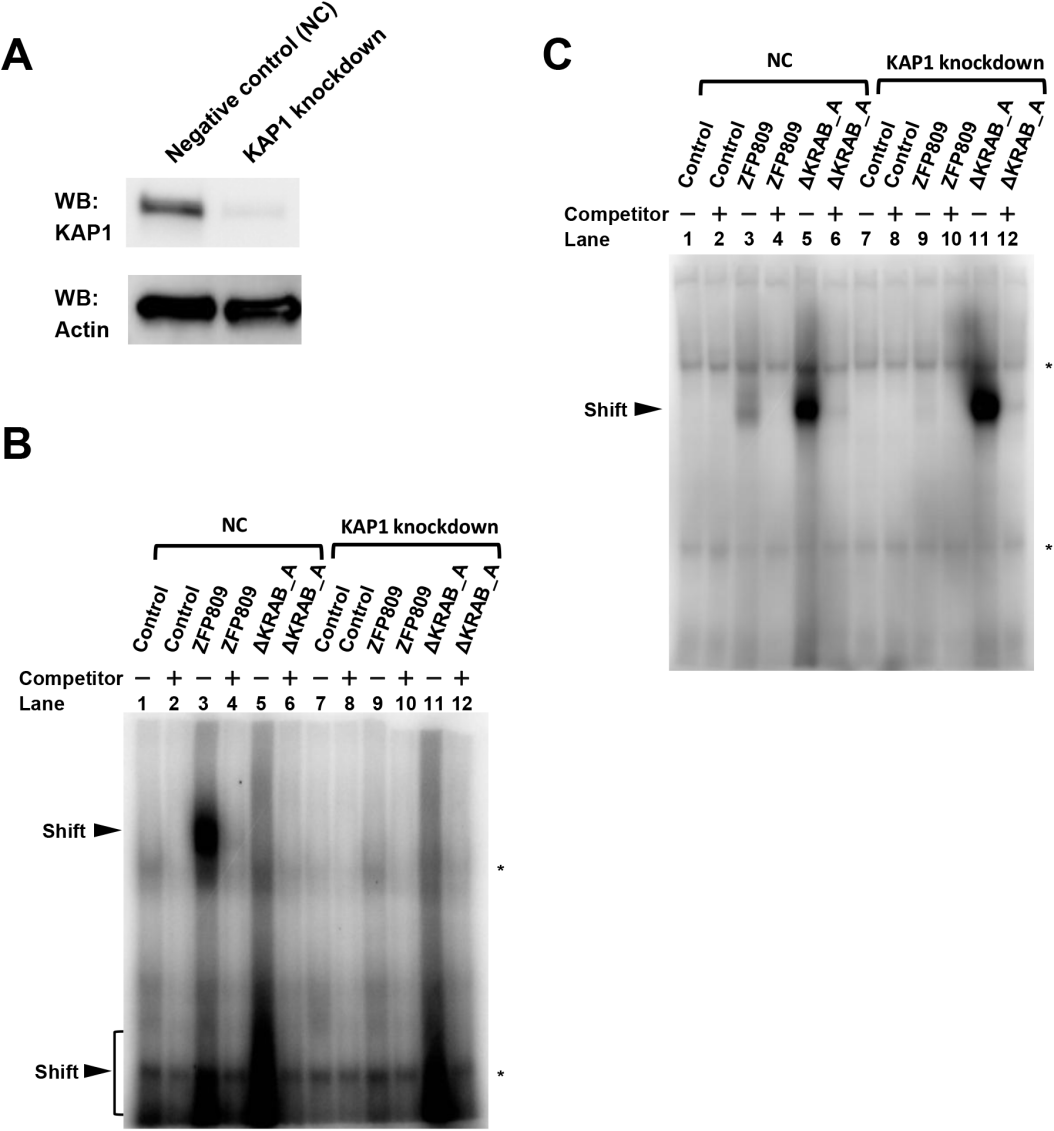
KAP1 dependent biding ability of ZFP809 to MLV-PBS. (A) Confirmation of knockdown efficiency for KAP1. 293FT cells transduced with lentivirus from shRNA vectors for NC or KAP1 were sorted based on EGFP expression. The sorted cells were subjected to Western blot analysis using anti-KAP1 and Anti-β-Actin antibodies. (B) and (C) EMSA of the MLV-PBS sequence with the intact ZFP809 and ΔKRAB_A proteins in the presence of cold competitor DNA on an 8% gel (B) and a 10% gel (C). Asterisks indicate non-specific bands.

We next examined whether the intact ZFP809 protein is able to bind to the PBS in the absence of KAP1. We established KAP1-knockdown (KD) 293FT cells using shRNA against KAP1 (Fig 5A). We transduced the KAP1-KD 293FT cells as well as control cells (293FT cells transduced with the negative control (NC) vector) with lentivirus vectors for the intact ZFP809 or ΔKRAB_A, and subjected the nuclear extracts of these cells to EMSA (Fig 5B and 5C).

**Fig 6:**
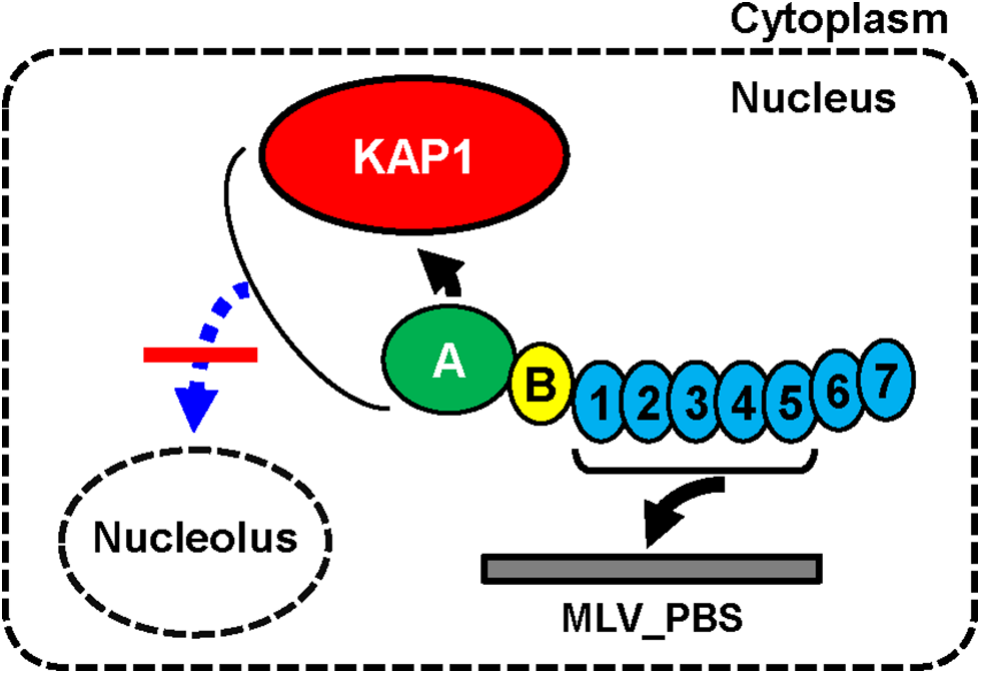
Models for ZFP809 sub-domains for nuclear localization, gene silencing and binding ability to MLV-PBS. ZFP809 contains a KRAB domain at the N-terminus and seven zinc fingers at the C-terminus. The nucleolar exclusion (under the nuclear localization) of ZFP809 is dependent of KAP1- KRAB_A domain interaction. Five (first to fifth) zinc fingers can bind to MLV-PBS in the absence of the KRAB_A domain (accordingly, without KAP1 interaction). However, the intact ZFP809 protein binds to MLV-PBS only when KAP1 interacts with the KRAB_A domain. Therefore, the interaction of KAP1 with the KRAB_A domain is essential for the PBS-dependent gene silencing primed by ZFP809.

Consistent with the EMSA results shown in Fig 4A, mobility shift of the probe DNA was observed for ZFP809 (Fig 5B, lane 3) and for ΔKRAB_A (lane 5) at upper and lower positions, respectively, when the nuclear extracts of NC cells were used. However, when the nuclear extracts of KAP1-KD cells were used, the mobility shift at the upper position for ZFP809 was lost (lane 9), while that at the lower position for ΔKRAB_A was observed (lane 11). The presence of the mobility shift at the lower position for ΔKRAB_A, irrespective of the type of nuclear extract (NC or KAP1-KD), was also confirmed by EMSA using a 10% gel (Fig 5C, lanes 5 and 10). These results demonstrate that ZFP809 binds to MLV-PBS only when KAP1 interacts with its KRAB_A domain, and further support the hypothesis that when the KRAB_A domain is free from KAP1, it prevents the zinc fingers of ZFP809 from binding to MLV-PBS (Fig 6 and S5 Fig). The results of EMSA experiments (Fig 4C and 4D and Fig 5B and 5C) suggest that the KRAB_A domain autoinhibits the binding of the zinc finger domain to the MLV-PBS when it is not bound to KAP1.

## Conclusions

We assessed the subcellular localization, gene silencing ability, and binding ability to PBS of a series of truncated and mutated ZFP809 proteins, and revealed the essential role of the KRAB A box for all functions assessed as well as the cooperative roles of a subset of zinc fingers. Our data also suggest that the interaction between KAP1 and the KRAB A box of ZFP809 potentiates KAP1-dependent gene silencing by ZFP809. Recent studies on KRAB-ZFPs indicate that the KRAB domain interacts with KAP1, and zinc fingers bind to a target sequence and also interact with proteins other than KAP1 [4]. As for ZFP809, EBP1 was recently identified a co-repressor, and was shown to be involved in triggering gene silencing [21]. Further characterization of ZFP809 sub-domains to determine regions responsible for interacting with EBP1 and other unidentified interacting partners will help us to elucidate the molecular mechanisms underlying gene silencing triggered by ZFP809.

## Supporting Information

S1 Fig Schemes of lentiviral and expression vectors. Structures of the lentiviral vector, pLVSIN_CMV/flag-X/IRES/mCherry (A) and the plasmid vector, pCMV/flag-X (B). One of the 11 cDNA fragments encoding full, truncated, and mutated ZFP809 proteins was inserted at downstream of the CMV promoter. X denotes either of the intact ZFP809, ΔSD, ΔZF, ZF1, ZF1-2, ZF1-3, ZF1-4, ZF1-5, ZF1-6, ΔKRAB_A, or mtKRAB.
(TIF)

S2 Fig Pairwise alignment of the amino acid sequences of ZNF268 and ZFP809 KRAB domains. The forty-five amino acid residues of the KRAB domain of the human ZNF268 (78 to 122 a.a. residues of NP_001159353) and the forty amino acid residues of the KRAB domain of the mouse ZFP809 (4 to 43 a.a. residues of NP_001158096) are aligned. Amino acid substitutions (E16/17A-W18A) in ZNF268 KRAB domain have been shown to abolish the interaction between the KRAB domain and KAP1 [18].
(TIF)

S3 Fig Co-localization frequencies of the intact, truncated, and mutated ZFP809 proteins with the fibrillarin (A) or KAP1 staining (B). We assessed whether the green fluorescent signal for a ZFP809 protein from (either intact, truncated, or mutated) in the nucleus is co-localized with the red fluorescent signal of the nucleolus marker, fibrillarin, to determine whether each of the ZFP809 protein forms is excluded from the nucleoli or not (A). We also assessed whether the nuclear localization pattern of a ZFP809 protein (excluded from the nucleoli or not) coincides with that of KAP1 (B). The frequency (%) of co-localization (A) or concordance of nuclear localization patterns (B) was determined by evaluating three independent sets of the images of 50 or more cells for each of the combinations of one of the ZFP809 proteins and with fibrillarin (A) or KAP1 (B). Vertical bars (with error bars) represent the mean percentages (with ± SD) of the cells in which co-localization (A) or concordance of the nuclear localization patterns (B) were observed.
(TIF)

S4 Fig Time course analysis of gene silencing by truncated and mutated ZFP809 proteins. 293FT cells transduced with one of the retroviral vectors (MLV/EGFP or MSCV/EGFP) were sorted based on EGFP expression, and transduced with one of the pLVSIN_CMV/flag-X/IRES/mCherry vectors (or used without lentiviral transduction as “Control”). Then, the expression levels of EGFP and mCherry were analyzed at day 4 (S4 Fig) and day 15 (Fig 3C) after transduction.
(TIF)

S5 Fig Models of the molecular actions of intact, truncated, and mutated ZFP809 on the MLV-LTR expression. This figure shows the models of ZFP809, ΔZF, ZF1-5, ΔKRAB_A or mtKRAB for gene silencing. ZFP809 binds to MLV-PBS through its zinc fingers, and interacts with KAP1, which further forms the silencing complex (HP1 and ESET are shown as members in the complex). This protein complex silences the MLV-LTR promoter activity. ZF1-5 can still bind to MLV-PBS, whereas ΔZF cannot. Therefore, only the former is able prime gene silencing. ΔKRAB_A binds to MLV-PBS but does not interact with KAP1. mtKRAB neither binds to MLV-PBS, likely due to autoinhibition for zinc fingers by the KRAB_A domain, nor interacts with KAP1 due to amino acid substitutions of E13A, E14A, and W15A. Both ΔKRAB_A and mtKRAB cannot recruit the silencing complex to the MLV-LTR promoter, thereby do not prime gene silencing.
(TIF)

S1 Table List of primers designed for vector construction. (XLSX)
