# Supplementary figures and images for: Functional Domains of ZFP809 Essential for Nuclear Localization and Gene Silencing
Source: PLoS One. 2015 Sep 29;10(9):e0139274. doi: 10.1371/journal.pone.0139274 (PMC4587795; doi:10.1371/journal.pone.0139274)

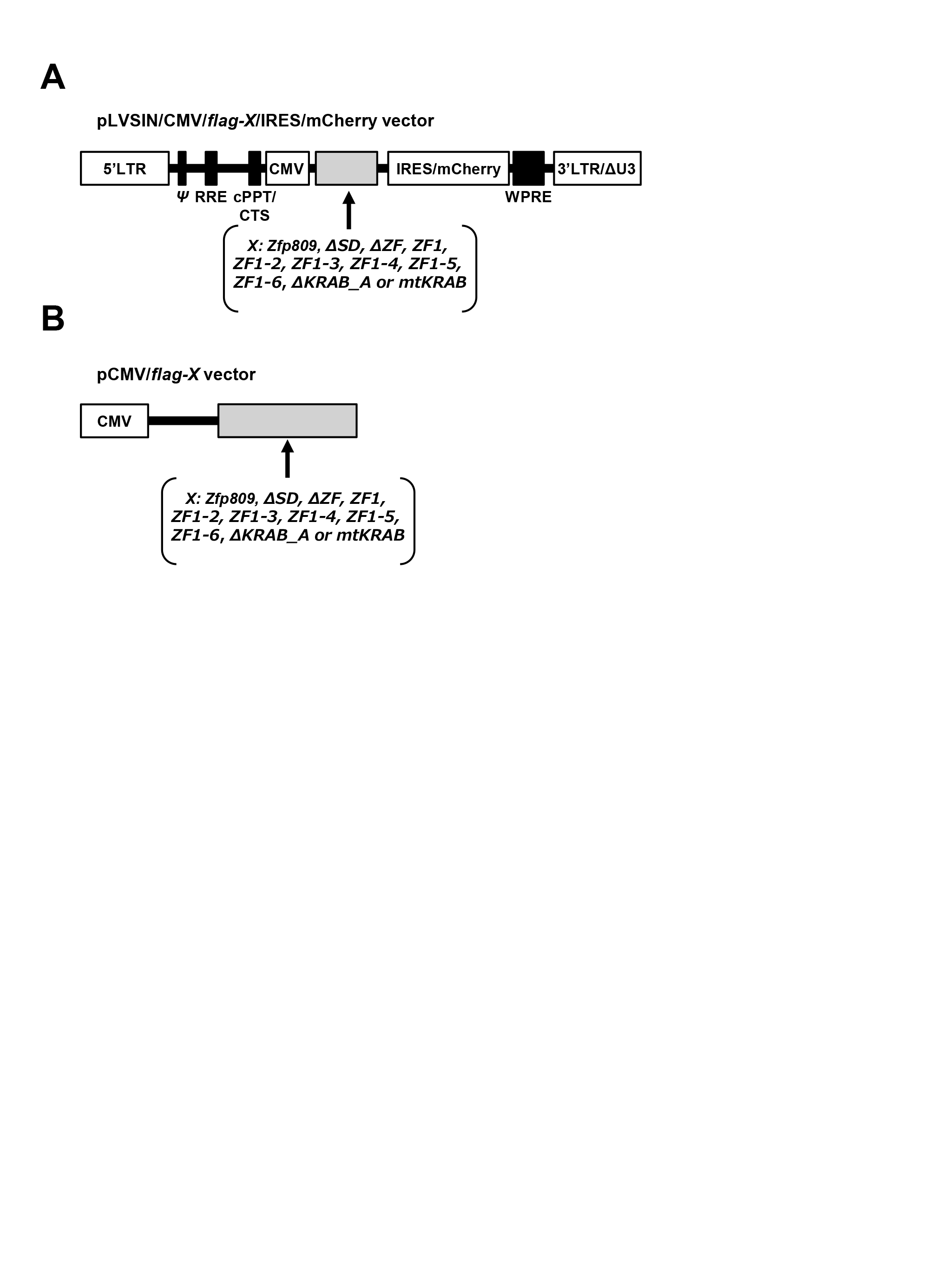

Supplement: S1 Fig — Structures of the lentiviral vector, pLVSIN_CMV/flag-X/IRES/mCherry (A) and the plasmid vector, pCMV/flag-X (B). One of the 11 cDNA fragments encoding full, truncated, and mutated ZFP809 proteins was inserted at downstream of the CMV promoter. X denotes either of the intact ZFP809, ΔSD, ΔZF, ZF1, ZF1-2, ZF1-3, ZF1-4, ZF1-5, ZF1-6, ΔKRAB_A, or mtKRAB. (TIF) [file pone.0139274.s001.tif]

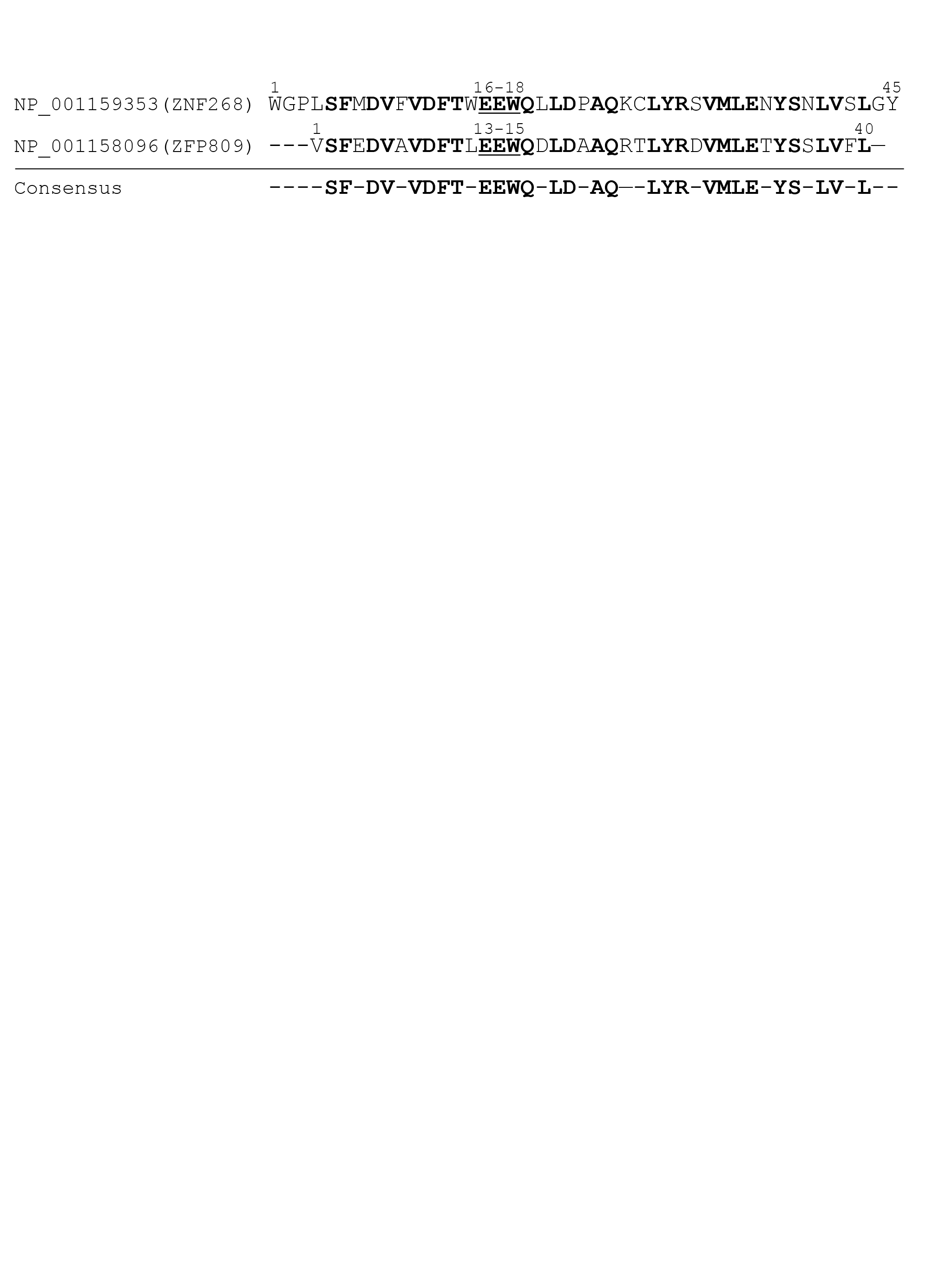

Supplement: S2 Fig — The forty-five amino acid residues of the KRAB domain of the human ZNF268 (78 to 122 a.a. residues of NP_001159353) and the forty amino acid residues of the KRAB domain of the mouse ZFP809 (4 to 43 a.a. residues of NP_001158096) are aligned. Amino acid substitutions (E16/17A-W18A) in ZNF268 KRAB domain have been shown to abolish the interaction between the KRAB domain and KAP1 [18]. (TIF) [file pone.0139274.s002.tif]

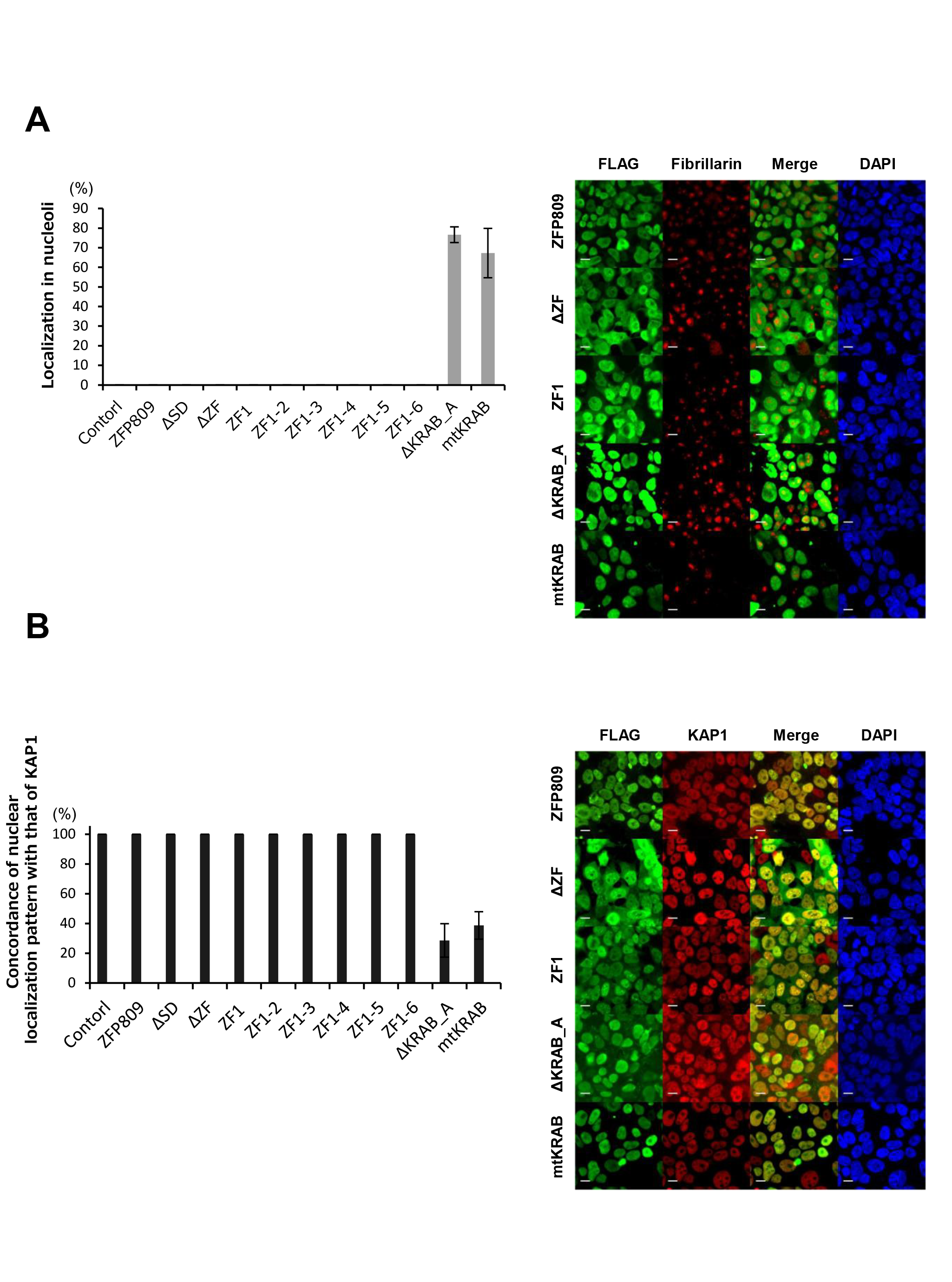

Supplement: S3 Fig — We assessed whether the green fluorescent signal for a ZFP809 protein from (either intact, truncated, or mutated) in the nucleus is co-localized with the red fluorescent signal of the nucleolus marker, fibrillarin, to determine whether each of the ZFP809 protein forms is excluded from the nucleoli or not (A). We also assessed whether the nuclear localization pattern of a ZFP809 protein (excluded from the nucleoli or not) coincides with that of KAP1 (B). The frequency (%) of co-localization (A) or concordance of nuclear localization patterns (B) was determined by evaluating three independent sets of the images of 50 or more cells for each of the combinations of one of the ZFP809 proteins and with fibrillarin (A) or KAP1 (B). Vertical bars (with error bars) represent the mean percentages (with ± SD) of the cells in which co-localization (A) or concordance of the nuclear localization patterns (B) were observed. (TIF) [file pone.0139274.s003.tif]

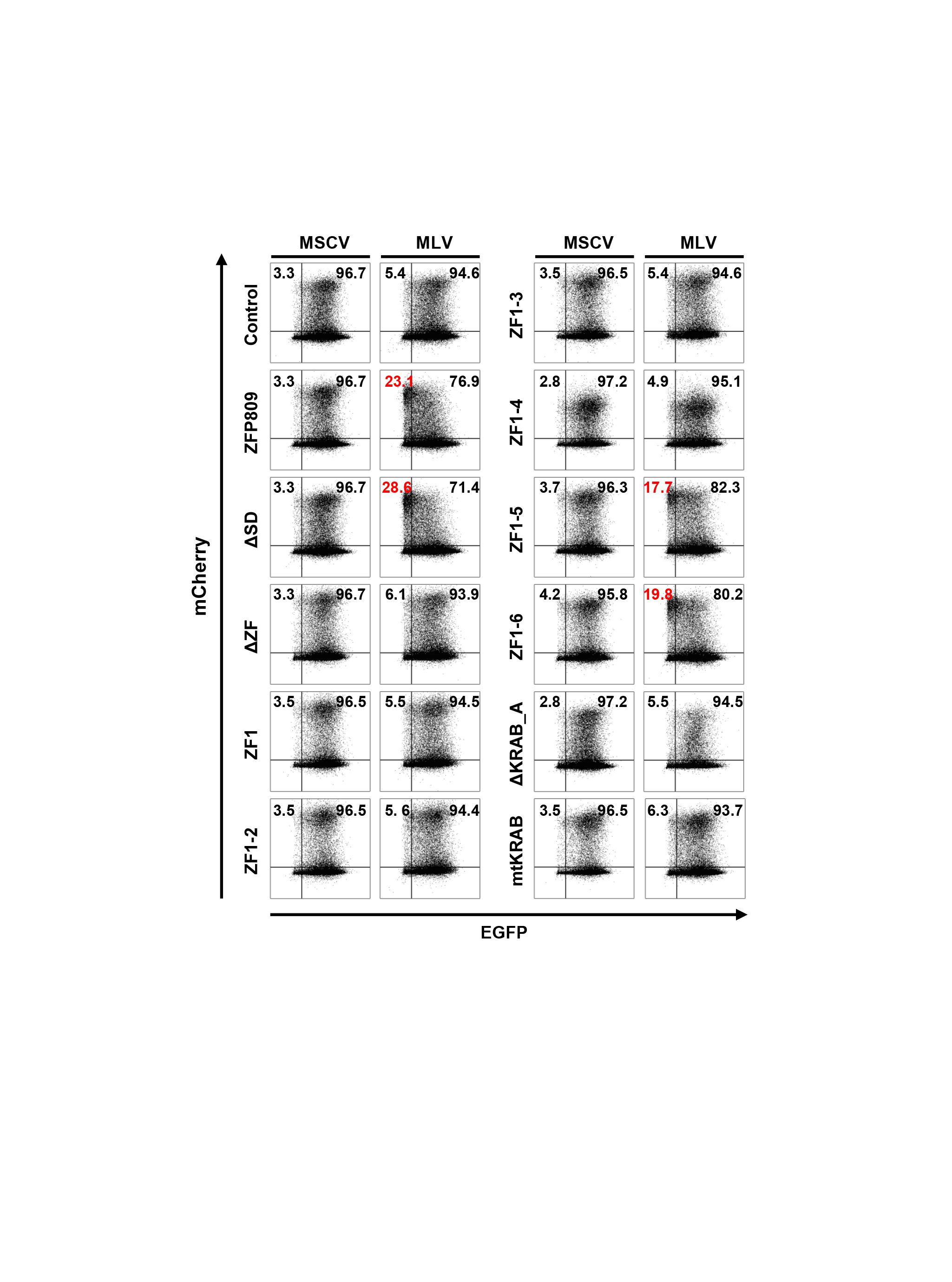

Supplement: S4 Fig — 293FT cells transduced with one of the retroviral vectors (MLV/EGFP or MSCV/EGFP) were sorted based on EGFP expression, and transduced with one of the pLVSIN_CMV/flag-X/IRES/mCherry vectors (or used without lentiviral transduction as “Control”). Then, the expression levels of EGFP and mCherry were analyzed at day 4 (S4 Fig) and day 15 (Fig 3C) after transduction. (TIF) [file pone.0139274.s004.tif]

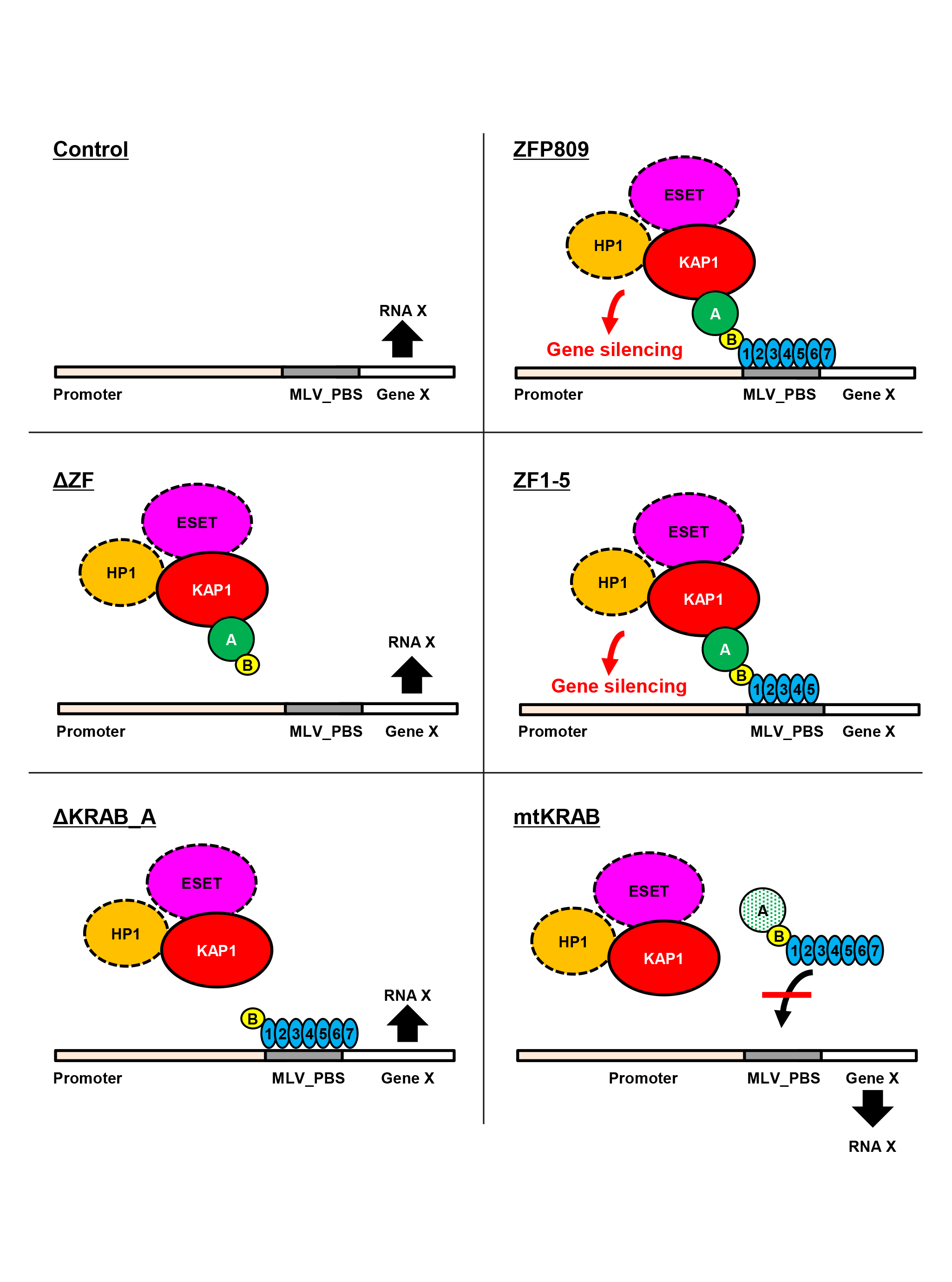

Supplement: S5 Fig — This figure shows the models of ZFP809, ΔZF, ZF1-5, ΔKRAB_A or mtKRAB for gene silencing. ZFP809 binds to MLV-PBS through its zinc fingers, and interacts with KAP1, which further forms the silencing complex (HP1 and ESET are shown as members in the complex). This protein complex silences the MLV-LTR promoter activity. ZF1-5 can still bind to MLV-PBS, whereas ΔZF cannot. Therefore, only the former is able prime gene silencing. ΔKRAB_A binds to MLV-PBS but does not interact with KAP1. mtKRAB neither binds to MLV-PBS, likely due to autoinhibition for zinc fingers by the KRAB_A domain, nor interacts with KAP1 due to amino acid substitutions of E13A, E14A, and W15A. Both ΔKRAB_A and mtKRAB cannot recruit the silencing complex to the MLV-LTR promoter, thereby do not prime gene silencing. (TIF) [file pone.0139274.s005.tif]
